# Promoting Antenatal Care Attendance Through a Text Messaging Intervention in Samoa: Quasi-Experimental Study

**DOI:** 10.2196/15890

**Published:** 2020-06-02

**Authors:** Jessica L Watterson, Diego Castaneda, Caricia Catalani

**Affiliations:** 1 School of Public Health University of California, Berkeley Berkeley, CA United States; 2 Design for Health IDEO San Francisco, CA United States; 3 Facebook Research Menlo Park, CA United States

**Keywords:** mHealth, antenatal care, maternal health, text messages

## Abstract

**Background:**

Antenatal care (ANC) has the potential to improve maternal health, but it remains underutilized and unevenly implemented in many low- and middle-income countries. Increasingly, text messaging programs for pregnant women show evidence that they can improve the utilization of ANC during pregnancy; however, gaps remain regarding how implementation affects outcomes.

**Objective:**

This study aimed to assess facilitators and barriers to implementation of an SMS text messaging intervention for pregnant women in Samoa and to assess its impact on ANC attendance.

**Methods:**

This study took place in Upolu, Samoa, from March to August 2014 and employed a quasi-experimental design. Half (n=3) of the public antenatal clinics on the island offered adult pregnant women the SMS text messaging intervention, with 552 women registering for the messages. At the comparison clinics (n=3), 255 women registered and received usual care. The intervention consisted of unidirectional text messages containing health tips and appointment reminders. The outcome of interest was the number of attended antenatal visits. Implementation data were also collected through a survey of the participating midwives (n=7) and implementation notes. Data analysis included a comparison of women’s baseline characteristics between the two groups, followed by the use of negative binomial regressions to test for associations between participation in the intervention and increased ANC attendance, controlling for individual characteristics and accounting for the clustering of women within clinics.

**Results:**

The comparison of ANC attendance rates found that women receiving the SMS text messaging intervention attended 15% fewer ANC visits than the comparison group (*P*=.004), controlling for individual characteristics and clustering. Data analysis of the implementation process suggests that barriers to successful implementation include women registering very late in pregnancy, sharing their phone with others, and inconsistent explanation of the intervention to women.

**Conclusions:**

These results suggest that unidirectional text messages do not encourage, and might even discourage, ANC attendance in Samoa. Interpreted with other evidence in the literature, these results suggest that SMS text messaging interventions are more effective when they facilitate better communication between patients and health workers. This study is an important contribution to our understanding of when SMS text messaging interventions are and are not effective in improving maternal health care utilization.

## Introduction

### Background

As an independent state, Samoa has achieved high performance on indicators of maternal health, including relatively high rates of deliveries in medical facilities (82%) and high rates of women receiving antenatal care (ANC, 93%) [1]. However, only 73% of women receive four or more antenatal visits, as recommended by the World Health Organization, and only 12% of women register for care in the first trimester [1]. To improve maternal health, rates of early, regular ANC attendance should be improved. Antenatal interventions, particularly those focused on chronic conditions (eg, anemia, infections, and hypertensive disorders), have the potential to detect, treat, or prevent conditions that could otherwise lead to maternal mortality or morbidity [2]. Samoa’s maternal mortality ratio was estimated at 100 maternal deaths per 100,000 live births in 2010 [3]. The Ministry of Health’s Antenatal Care Survey in 2012 found that many mothers did not think they needed to attend ANC because they felt their baby was safe and in good health (23%; Samoa Ministry of Health, unpublished data, 2012). These results indicate that the importance of ANC must be emphasized to pregnant women to ensure they attend ANC, even if they feel healthy.

Text message reminders and education interventions for pregnant women have been implemented widely around the world, but relatively few have been systematically evaluated to determine their effects on maternal care-seeking behavior or health outcomes. Among the studies that have examined outcome measures, there is some evidence that SMS text messaging programs can improve health care utilization, knowledge, and satisfaction with care. For example, Lund et al [4] conducted a pragmatic randomized controlled trial (RCT) in Zanzibar and found that women receiving unidirectional text messages and free mobile phone credit to communicate with their health provider had double the odds of attending four or more antenatal visits, relative to the control group. Similarly, studies in Malawi and Iraq found increased ANC attendance among women who received a unidirectional text or voice messaging intervention and access to hotlines or phone numbers to call with questions [5,6]. A recent meta-analysis of seven RCTs in low- and middle-income countries found evidence that text messages for pregnant women significantly increased ANC attendance by 174% [7]. Other studies have also found SMS text messaging interventions to increase mothers’ knowledge, preparedness, feelings of empowerment, and satisfaction with ANC [7-10].

Samoa provides a promising context in which to study text messages for maternal health because an estimated 90% of the population of Samoa had access to a mobile phone in 2013 [11], and nearly 99% of the adult population is literate [3]. In addition, free ANC is provided at public health facilities across the country. Although a handful of studies have found evidence for the effectiveness of SMS text messaging programs at increasing ANC attendance, more evidence is needed to understand in what environments these programs can produce results for women’s health [12]. Previous studies have examined the outcomes of these programs in countries in Africa and Asia with different cultures, religions, literacy rates, incomes, and health care systems—all factors that could contribute to or detract from the effectiveness of a pregnancy SMS text messaging intervention. Therefore, this study explores whether this intervention can be effective in the Samoan context, contributing to a more nuanced understanding of how the setting and implementation factors might affect the outcomes of a pregnancy SMS text messaging program.

### Hypotheses

On the basis of earlier findings that SMS text messaging interventions have been successful at improving ANC attendance in other developing countries, we hypothesized the following:

1. Pregnant women receiving the SMS text messaging intervention will attend a higher number of follow-up antenatal visits than women not receiving them, controlling for other individual characteristics;
2. The SMS text messaging intervention will have a greater effect on younger pregnant women’s ANC attendance compared with older women, controlling for other individual characteristics.

Evidence from around the world indicates that younger people tend to have higher rates of mobile phone ownership and higher technological literacy [13,14], suggesting that the effect of an SMS text messaging intervention could be even greater for younger women. In addition, these women are more likely to be first-time mothers and to be interested in additional supportive information, such as that provided by the SMS text messaging program.

## Methods

### Study Design

This study was conducted from March to September 2014 in Samoa. The study took place on the island of Upolu, the most populated island and home to the capital city, Apia. The National Health Service runs 6 health centers on the island (1 urban and 5 rural), all offering free antenatal services to pregnant women. Ethics approval for this study was obtained from the National Health Research Committee of Samoa on February 6, 2014. Per the approved protocol, participants receiving the intervention provided verbal consent to participate in the study to the clinic midwives. Analysis of the deidentified dataset was deemed to be *not human subjects research* by the University of California Berkeley Office for the Protection of Human Subjects on September 7, 2017.

**Figure 1:**
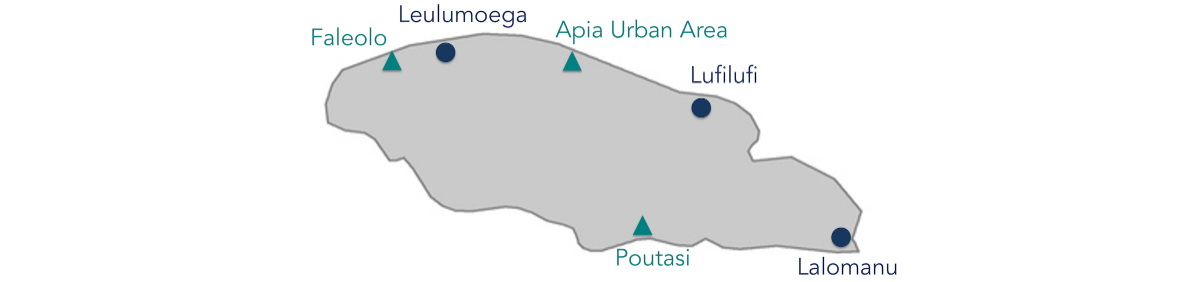
Map of National Health Service clinics in Upolu, Samoa. Circles indicate a comparison clinic and triangles indicate an intervention clinic.

This study employed a cluster-randomized quasi-experimental study design, in which half of the health centers (n=3) were randomly selected to offer the SMS text messaging intervention to pregnant women presenting for their first antenatal visit, and the other half of the clinics (n=3) were randomly selected to offer the usual care only. Random selection was performed by assigning each clinic a number from 1 to 6 and then using a Web-based random number generator to select 3 of the numbers randomly to identify intervention clinics. Figure 1 illustrates the locations of these clinics on Upolu Island. Nurse-midwives offered the SMS text messaging intervention to pregnant women (n=728) who registered at an intervention clinic. Pregnant women who registered at comparison clinics during the study period were enrolled in the comparison group (n=251).

The study included a total of 979 women, all of whom registered at 1 of the 6 public antenatal clinics in the study period. The only pregnant women not eligible for inclusion in the study during this period were those who did not attend ANC in a clinic (eg, those visiting a traditional birth attendant, estimated at 3% of pregnant women [1]), or those who visited a private health care provider. This is a relatively small percentage of the population, on the basis of the significantly higher cost and limited reach of most private facilities (most are located in the capital city, Apia).

Women in the intervention group received 2 educational messages per week, with content adapted to their gestation (eg, if a woman was 20 weeks pregnant at registration, the first educational messages she received were adapted for 20 weeks of pregnancy, then 21 weeks the following week, and so on). All women in the intervention group received the same educational messages at the same gestational age (ie, the messages were not tailored to individuals). The text messages were adapted for the local context and translated to Samoan from the free library developed by the Mobile Alliance for Maternal Action on the basis of the Lancet Maternal and Neonatal Survival Series. Adaptations included removing content about malaria (malaria is not endemic in Samoa) and ensuring fruits and vegetables that were referenced were familiar and locally available. Women in the intervention group also received a text message appointment reminder the day before their scheduled appointment. Finally, a reminder message was sent to women who were over 4 weeks overdue for an appointment.

This study examined the effect of text message education and reminders on ANC attendance, which was measured by the number of follow-up ANC visits attended. This outcome was selected on the basis of earlier research that found SMS text messaging programs showed promise for improving ANC attendance in other settings [4-6]. Data were collected from medical records and ANC registration books in antenatal clinics. Although women were of different gestations at registration, and therefore had different recommended antenatal schedules, gestational age was controlled for in the multivariate analyses. All available demographic information was also collected from medical records for each woman, including her age, marital status, parity, and whether she or her partner was employed outside the home and her home village. These demographic details are comparable with those included in similar studies and are thought to be potential confounders for ANC attendance, which is why they were included in the analysis. Details on the variables collected for both the intervention and control groups are outlined in Table 1. A survey for implementation feedback was also conducted about 1 month after beginning the program with the implementing midwives (n=7), and the researcher maintained detailed implementation notes and records.

**Table 1:** Description of variables and data sources.

Variable	Description	Data source
Age	Age in years at time of ANC^a^ registration	Medical record or ANC registration book
Parity (including current pregnancy)	Total number of pregnancies, including current pregnancy	Medical record
Distance from home village to registration clinic (km)	Distance from home village to the clinic where the woman registered for ANC in kilometers (km)	Home village recorded from medical record or ANC registration book, then distance from the registration clinic in km was estimated using Google Maps
Married/in partnership	Marital status recorded as married or stable union	Medical record
Employed and/or partner employed	Occupation of the pregnant woman and/or husband/partner was recorded, then categorized as being at home or outside the home. If 1+ person worked outside the home, they were categorized as employed	Medical record
Gestation at registration (weeks)	Number of weeks pregnant at the time of registration for ANC	Medical record or ANC registration book
Number of follow-up antenatal visits attended	Number of visits attended after the first registration visit; dates of subsequent visits were recorded, then counted	Medical record or ANC registration book
Intervention group	Enrolled in the intervention group if women were pregnant, over 18 years of age, presented to an intervention clinic for ANC registration, and agreed to participate	Sign-up sheet or registration book from midwives in clinic

^a^ANC: antenatal care.

The required sample size was estimated first without accounting for clustering, as in similar studies [4]. To detect a difference of one follow-up antenatal visit between the intervention and control groups with alpha=.05 and beta=.10, a sample size of 262 is needed (n=131 per group). This estimate was on the basis of a conservative approximation of the effect size and standard deviation found in a study by Alhaidari et al [6], which also examined the effect of an SMS text messaging intervention on the number of ANC visits attended. Although this estimate did not take clustering into account, it was known that the final sample size would be significantly larger given Samoa’s birth rate, the population of Upolu, the length of the study, and the high percentage of pregnant women who attend at least one ANC visit with a health care provider [1].

### Missing Data

Problems with locating complete paper medical records led to one or more missing demographic variables for 214 participants. Varied filing systems, large volumes of patients seen each day, and many common names led to difficulty locating patients’ records, both for the researcher and for clinic staff. The distribution of this missing data is outlined in Table 2 in the Results section. The missing data were relatively evenly distributed across both intervention and comparison groups, reducing concerns about bias. The main analyses used listwise deletion of these observations with missing values (106 from the per-protocol intervention group and 108 from the per-protocol comparison group). As a sensitivity analysis, multiple imputations by chained equations was used to generate 20 datasets with 975 complete observations each, which were then combined for analysis using Rubin combination rules [15]. Data were imputed for the variables age at registration, parity, marital status, and employment status. Data were not imputed for the 4 observations missing the distance from their home village to the registration clinic because of the high correlation of this variable with other variables (the model did not achieve convergence). The number of imputed datasets was determined using the proportion of missing data and acceptable power falloff [16]. The sensitivity analysis then proceeded with the same models as the main analysis (described below in the Data Analysis section), and the results were compared.

### Data Analysis

Statistical analyses were conducted using the Stata/SE 13.0 software (Stata Corp LP). Basic descriptive statistics were calculated for all variables and separately for the intervention and comparison groups. This included means, medians, and standard deviations for all continuous variables and frequencies, proportions, and 95% CIs for all categorical variables. Descriptive statistics for both groups were compared using *t* tests for continuous variables and chi-square tests for categorical variables.

The intervention and comparison groups were categorized using both the intention-to-treat and per-protocol principles. In the intention-to-treat analysis, all women registering for ANC at an intervention clinic were treated as receiving the intervention, regardless of whether they signed up to receive the text messages or not. In the per-protocol analysis, the women who did not actually receive any text messages were considered part of the comparison group, regardless of at which clinic they registered.

To study the significance of differences in the number of antenatal visits that were attended between the two groups, negative binomial regressions were estimated, controlling for patient demographics and accounting for clustering within clinics using a clustered sandwich estimator to produce robust standard errors. Next, the same model was run with an interaction term for young women (defined as under the age of 25) and being in the intervention group.

Implementation survey data were analyzed by calculating basic descriptive statistics for quantitative questions. Open-ended responses to survey questions and implementation notes were carefully reviewed to identify common themes.

## Results

### Descriptive Statistics

**Figure 2:**
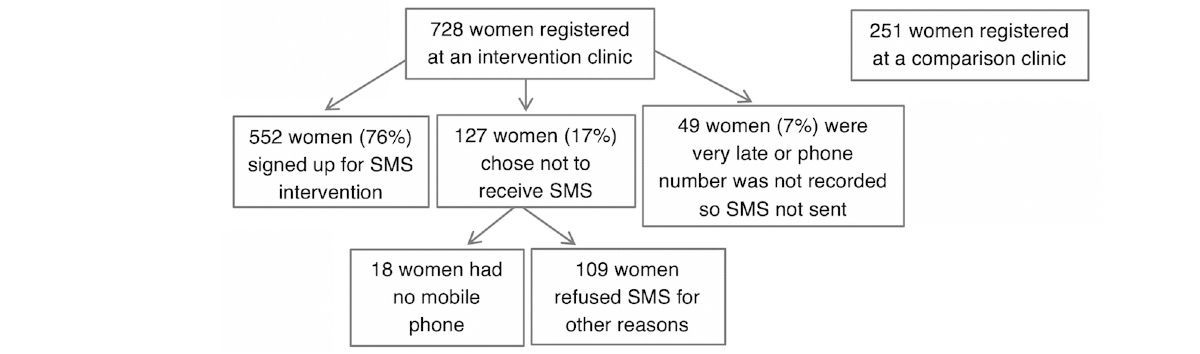
Antenatal care and text message (SMS) intervention registration results (N=979).

Figure 2 outlines the results of the study registration. A total of 728 women registered for ANC at 1 of the 3 intervention clinics during the study period. Of these women who were offered the SMS text messaging intervention, 75.8% (552/728) signed up. The majority of women who registered at an intervention clinic but did not receive the text messages registered very late in pregnancy (ie, within 2 weeks of their due date), or their phone number was not recorded so messages could not be sent (n=49). A total of 127 women elected not to receive the text messages, and 18 of those women did not have a mobile phone. A total of 251 women were registered at a comparison clinic during the study period.

Challenges locating complete paper records led to one or more missing demographic variables for 214 participants. The distribution of this missing data is outlined in Table 2. These observations with missing values were excluded from subsequent analyses.

**Table 2:** Distribution of missing observations across groups.

Variable	Intention-to-treat, n (%)		Per-protocol, n (%)	
	Intervention (n=728)	Comparison (n=251)	Intervention (n=552)	Comparison (n=427)
Age	128 (17.6)	45 (17.9)	95 (17.2)	78 (18.3)
Parity (including current pregnancy)	127 (17.4)	47 (18.7)	97 (17.6)	77 (18.0)
Distance from home village to registration clinic (km)	4 (0.5)	0 (0.0)	1 (0.2)	3 (0.7)
Married/in partnership	94 (12.9)	42 (16.7)	66 (12.0)	70 (16.4)
Employed and/or partner employed	147 (20.2)	61 (24.3)	104 (18.8)	104 (24.4)
Missing any of the above variables	151 (20.7)	63 (25.1)	106 (19.2)	108 (25.3)

Descriptive statistics for both intervention and comparison groups with complete data according to both intention-to-treat and per-protocol categorization are outlined in Table 3. The size of the intervention group was larger than that of the comparison group because of the inclusion of the antenatal clinic in the main hospital as an intervention site (Tupua Tamasese Meaole Hospital). This clinic saw the highest number of women registering for ANC, which resulted in the larger intervention group. The demographic characteristics of women in the intervention and comparison groups were similar at baseline, with two exceptions. The proportion of women and/or their partners who were employed outside the home was significantly higher in both intervention groups, regardless of whether they were categorized according to per-protocol or intention-to-treat. Similarly, the mean distance traveled by women from their home village to the clinic they registered at was higher among the intervention groups than in the comparison groups, although these distances varied widely (from 0 to 117 km), and thus have high standard deviations. This was also likely because of the inclusion of the main hospital as an intervention site, as women are more likely to have traveled from a rural area to the capital city to attend their appointment there.

Women in the per-protocol intervention group received a mean of 25.6 messages throughout the intervention (SE 0.47), and women in the intention-to-treat intervention group received a lower mean of 19.3 messages (SE 0.54) because of the fact that 176 of these women received no messages, despite being randomized to receive them (results not shown in the table).

**Table 3:** Baseline characteristics of intervention and comparison groups.

Variable		Intention-to-treat			Per-protocol		
		Intervention (n=577)	Comparison (n=188)	*P* value	Intervention (n=446)	Comparison (n=319)	*P* value
**Continuous variables, mean (SD)**							
	Age	26.7 (6.4)	27.1 (6.5)	.53	26.6 (6.3)	27.2 (6.5)	.18
	Parity (including current pregnancy)	3.2 (2.0)	3.3 (2.0)	.62	3.1 (1.9)	3.3 (2.1)	.25
	Distance from home village to registration clinic (km)	11.9 (13.1)	6.6 (7.2)	<.001	12.3 (13.9)	8.3 (8.6)	<.001
	Gestation at registration (weeks)	27.2 (6.7)	26.5 (6.0)	.13	27.4 (6.5)	26.6 (6.6)	.10
	Number of follow-up antenatal visits attended	2.2 (1.9)	2.6 (1.7)	.01	2.1 (1.7)	2.5 (1.9)	<.001
**Categorical variables, n (%)^a^**							
	Married/in partnership	519 (89.9)	171 (91.0)	.69	401 (89.9)	289 (90.6)	.75
	Employed and/or partner employed	405 (70.2)	89 (47.1)	<.001	327 (73.3)	167 (51.9)	<.001

^a^Excluding missing data.

### Comparison of Antenatal Care Visits Attended for Intervention and Comparison Groups

Using the intention-to-treat principle, women registering at intervention clinics attended, on average, only 2.2 follow-up visits, as compared with 2.6 in the comparison group (*P*=.01). Similarly, in the per-protocol analysis, women receiving the intervention attended only 2.1 follow-up visits on average, compared with 2.5 visits in the comparison group (*P*<.001). These unadjusted comparisons are presented in Table 3.

Contrary to hypothesis 1, the negative binomial regression analyses (Table 4) showed that women in the intervention group attended 13% (intention-to-treat) to 15% (per-protocol) fewer follow-up ANC visits than women in the comparison group, controlling for all covariates. The interaction term between younger women (defined as under 25 years old) and receiving the intervention in the subsequent regression model was not significant (*P*=.30), suggesting that the effect of the intervention on ANC attendance was similar across age groups (results not shown in table). Therefore, support was not found for hypothesis 2.

As a sensitivity analysis, the multivariate regressions were run again with the 20 multiply imputed datasets (n=975). The estimated effect of receiving the intervention on the number of follow-up ANC visits attended was slightly smaller (ie, the incidence rate ratio [IRR] was closer to 1.0) and no longer statistically significant in these regression results (IRR=0.88, *P*=.06, per-protocol).

**Table 4:** Comparison of visits attended between intervention and comparison groups, controlling for demographic characteristics.

Variable	Intention-to-treat			Per-protocol			
	IRR^a^	Robust SE	*P* value		IRR	Robust SE	*P* value
Intervention group	0.87	0.06	.07		0.85	0.05	.004
Age at registration	1.01	0.01	.48		1.01	0.01	.50
Married/in partnership	0.95	0.10	.63		0.95	0.11	.67
Parity	0.98	0.02	.39		0.98	0.02	.37
Employed and/or partner employed	0.91	0.06	.15		0.92	0.07	.26
Distance from home village to registration clinic (km)	1.00	0.00	.38		1.00	0.00	.30
Gestation at registration (weeks)	0.99	0.00	.001		0.99	0.00	.004
Constant	3.36	0.58	<.001		3.31	0.52	<.001

^a^IRR: incidence rate ratio.

### Midwife Survey and Qualitative Results

The survey of implementing midwives (Table 5) indicated that they found the program to be useful (mean score of 4.0 out of 5). The average rating of how interested they thought their patients were in receiving the messages was lower (mean score of 3.1 out of 5) compared with other questions. In addition, the midwives felt that registering pregnant women for the messages (which involved recording the woman’s name, phone number, and gestation on a form) was fairly difficult (mean score of 4.29 out of 5, where 5 is difficult).

**Table 5:** Quantitative results of survey of implementing midwives (N=7).

Question	Score, mean (SD)
(1) Please rate how easy or difficult it is to register pregnant women for the text messages on a scale of 1 to 5 (1=easy, 5=difficult)	4.29 (0.76)
(2) Please rate how interested you think your patients are in receiving text messages during their pregnancy on a scale of 1 to 5 (1=not interested, 5=very interested)	3.14 (1.86)
(3) Please rate how useful you think this text message program is for your patients on a scale of 1 to 5 (1=not useful, 5=very useful)	4.00 (1.83)

Analyses of qualitative data from implementing midwives and implementation notes identified facilitators and barriers to a successful implementation of the SMS text messaging program. A key barrier was difficulty with consistently offering and explaining interventions to women at intervention clinics. Despite the implementing midwives participating in training at the program’s start and regular visits from the researcher to discuss the program and collect data, evidence suggests that some pregnant women might not have received a clear explanation of the program, or might not have been offered the program even if they registered for ANC at an intervention clinic. One midwife wrote, “[I] sometimes forget to fill in forms but will improve as it becomes part of daily routine.” This quote highlights that implementation of the intervention did not fit into the midwives’ existing workflow, which might have contributed to inconsistent registration, and could explain why the midwives rated registering pregnant women for the program as fairly difficult. In addition, the researcher received responses to some of the text messages asking who had sent the message. This could suggest potential issues such as (1) someone else was using the mobile phone, as phone sharing is a common practice among friends and families in Samoa, or (2) the woman had not understood or had forgotten that she signed up for the messages at the clinic.

One of the key facilitators identified was offering the option for women to enroll in the message by paper during their ANC visit, rather than requiring them to send an SMS text message to enroll. Many mobile messaging platforms enroll participants by having them send a short code to a phone number. However, this can cost the participant’s mobile phone credit to send a message. All but one of the participants in this study chose to enroll by paper, suggesting that it was the preferred enrollment option in this population.

Implementing midwives also suggested ways to improve the program if it were to be continued, and 2 midwives suggested adding messages telling pregnant women to avoid abdominal massage during their pregnancy, as massage is a common practice by traditional healers in Samoa. One midwife also suggested trying to get husbands or partners to participate in the SMS text messaging program.

## Discussion

### Principal Findings

Despite some previous evidence for the effectiveness of SMS text messaging interventions for increasing attendance to antenatal visits, our results indicate that they may not necessarily be effective at improving health-seeking behavior when implemented in isolation of other interventions, such as hotlines or phone credit, to ask questions. In fact, this study found some evidence that women receiving the unidirectional messages attended fewer follow-up ANC visits than did women not receiving the messages, controlling for individual characteristics and clustering within clinics. One potential explanation for this finding could be that the messages led participants to feel more connected to the clinic, or that they had sufficient information, reducing their motivation to attend an in-person check-up (ie, there was a substitution effect, whereby patients substituted information received by text message for more time-intensive ANC). Further study is needed to understand the components of SMS text messaging programs that encourage (or discourage) ANC attendance and whether adjustments to the implementation (eg, features, content, and scheduling) could impact the effectiveness of the intervention in improving attendance.

### Comparison With Prior Work

To date, only a handful of studies have examined outcome measures for SMS text messaging interventions for maternal health and found positive results, and each of these studies included some features beyond what our intervention offered [12]. For example, a study in Sierra Leone found an increase of 11.3% in attendance at the fourth antenatal visit after implementation of a bidirectional SMS text messaging intervention that allowed pregnant women to communicate with health care workers [17]. Similarly, a study in Malawi found an increase in antenatal attendance after implementing a case management hotline and unidirectional text and voice messaging [5]. A recent literature review of studies using SMS text messaging for maternal and infant health found evidence that bidirectional messaging might be more effective [18]. Taken together, previous evidence and our study indicate that interventions may need to increase bidirectional interaction with pregnant women and move beyond unidirectional reminders and health tips. Enhancing patient engagement may enable text-based interventions to have a greater impact on patient care-seeking behavior. On the basis of our implementation findings, another idea to explore in future research is whether the participation of women’s partners, family members, or friends could improve the program’s outcomes. A recent meta-analysis of male involvement during pregnancy found evidence of improved utilization of maternal health services [19], lending further support to the idea that women’s social networks could support them to attend more antenatal visits if involved in the SMS text messaging program.

### Limitations

Digital intervention research is still in its infancy, especially in the developing world, and as such, there were limitations that may have affected the effectiveness of this program. Future studies should take these into account to continue to improve our understanding of these interventions. First, cluster randomization (at the health center level) was preferred because it did not require health workers to keep track of which individuals received the intervention. In doing so, we were unable to randomize individual women to the intervention, which would have improved causal inference.

Second, data on the presence of pregnancy complications were not available. Pregnancy complications could have influenced the number of ANC appointments attended (eg, if a woman is experiencing complications, her midwife will encourage her to come for more frequent check-ups). However, women were assigned to the intervention or comparison group on the basis of which clinic they first presented to, regardless of later transfers to the main tertiary hospital in Apia (as would occur if a complication was identified). This suggests that complications might have also been evenly distributed across groups. However, we have no way to test this with our current dataset. Further, if any bias were introduced by the presence of more complications in one group, we would expect that women with more complications would have presented to the main tertiary hospital, which was an intervention site. Therefore, this would have likely biased our results such that the intervention group would have attended more visits than the comparison group (opposite to our findings).

Third, one intervention clinic was based at the main hospital in the capital city and thus was significantly larger than any of the other clinics. Women traveled from many rural parts of the island to receive ANC at this clinic, but we do not know if there are other systematic ways in which the women registering at this clinic are different from women registering elsewhere. We account for the potential of longer distances traveled and the clustering of women within clinics in the multivariate regression models in an attempt to address this issue.

Fourth, because of difficulties in locating paper medical records in many of the clinics, there was a significant amount of missing demographic data. Attendance data for these patients were still collected from registration books, so only demographic data were missing. The results of the sensitivity analysis with multiply imputed data found that the intervention and comparison groups attended a similar number of follow-up ANC visits, which could suggest that the lower attendance found in the intervention group in the main analysis could have been because of bias introduced by the missing demographic data.

Finally, the surveys were completed by a relatively small number (n=7) of midwives who were directly involved in the program, and therefore may not be representative of the views of all clinic staff involved in ANC. Future research should also collect feedback directly from pregnant women participating in the intervention to identify other areas for improvement.

### Conclusions

When combined with the other limited findings available on SMS text messaging interventions for ANC, the level of interaction between women and the program may explain differences in the effectiveness of interventions. More information will not necessarily increase care-seeking behavior—it could deter women from attending antenatal visits. This intervention was relatively low intensity and likely was not sufficient to overcome larger barriers to women seeking ANC, such as transportation, inconvenience, competing priorities, and cultural factors. Further study of the specific features of SMS text messaging programs for pregnant women that contribute to their effectiveness should be a high research priority.

## Abbreviations

ANC: antenatal care
IRR: incidence rate ratio
RCT: randomized controlled trial
